# Development of Polyps and Cancer in Patients with a Negative Colonoscopy: A Follow-Up Study of More Than 20 Years

**DOI:** 10.1155/2014/261302

**Published:** 2014-03-04

**Authors:** R. J. L. F. Loffeld

**Affiliations:** Department of Internal Medicine and Gastroenterology, Zaans Medisch Centrum, Postbus 210 1500 EE Zaandam, The Netherlands

## Abstract

*Background.* Adenomas are missed during colonoscopy. *Aim.* Assess the occurrence of colorectal cancer (CRC) and polyps in patients with a negative index colonoscopy (IC). *Patients and Methods.* All patients with a IC in 1992–1994, aged 40 and 60 years, were included. Exclusion criterion was presence of abnormalities, a family history, or surveillance. At the end of 2013 all records were studied in order to gather follow-up information. *Results.* 394 patients were included in four groups: group 1 patients who died, group 2 patients who were not in the hospital systems anymore, group 3 patients still visiting the hospital but not the department of gastroenterology, and group 4 patients undergoing new colonoscopies. In group 1, 2 patients died of CRC and 4 developed a polyp. No data were available from the patients in group 2. Patients in group 3 visited the outpatient clinics but did not undergo new colonoscopy. Patients in group 4 underwent additional colonoscopies. The yield was 35 patients polyps and three CRCs. Five patients (1.3%) developed CRC, and 39 (9%) developed a polyp. *Conclusion.* Given these results the number of potentially missed adenomas in IC is very low and the consequences of missed adenomas are highly exaggerated.

## 1. Introduction

According to the literature many adenomas are missed during routine endoscopic investigation of the colon [1, 2]. This leads to the assumption that many potential future cancers are being missed. The miss rate of adenomas is reported to exceed 20% in back to back endoscopic investigations. Is this miss rate indeed responsible for interval cancers? Obviously, the answer to this question is yes. This will certainly be the fact in patients with positive family history of colorectal cancer and patients belonging to families with the Lynch syndrome [3]. Is this also true for patients with a normal risk of developing colorectal cancer? Or will all these missed adenomas eventually become cancer? Is the risk of development of colorectal cancer due to missed adenomas exaggerated in normal daily practice [4]?

In order to try to answer these questions, a study was done in patients who underwent colonoscopy for the normal obvious clinical reasons, in whom no significant findings were diagnosed, in order to ascertain the development of polyps and colorectal cancer in many years to follow.

## 2. Patients and Methods

All patients undergoing colonoscopy in the years 1992–1994 in the Zaans Medisch Centrum, the community hospital of the Zaanstreek region in the Netherlands, were studied. Patients underwent endoscopy for normal clinical reasons like abdominal complaints, anaemia, rectal bleeding, and so on.

Primary inclusion criterion was the age of the patients at time of the colonoscopy. This had to be between 40 and 60 years; this is the age in which adenomas are mostly diagnosed for the first time. The secondary inclusion criterion was the presence of a negative colonoscopy. This is absence of any clinically important findings with the exception of diverticuli and haemorrhoids. Exclusion criteria were presence of a family history of colorectal cancer (including the Lynch syndrome), presence of polyps, colonoscopy done because of follow-up after prior removed adenomas (hence surveillance endoscopies), presence of colorectal cancer, and prior colorectal cancer, and inflammatory bowel disease.

The procedure was done with Olympus fibre-optic and video endoscopes (EVIS 100) after normal standard colon cleansing.

At the end of the year 2013 all hospital records were studied in order to gather information on all included patients. The records of the hospital, the records of the endoscopy department, and pathology records were studied. The primary goal was to assess whether patients underwent new colonoscopy in the years following 1992–1994 and whether polyps or colorectal cancer were detected. Cause of death was determined if possible.

In addition, especially if patients were not registered in the hospital system anymore, the national data registry on pathology (PALGA) was consulted in order to determine whether adenomas or colorectal cancer was diagnosed in these patients in other hospitals in the Netherlands.

Statistical analysis was done with chi-square test for contingency tables. A value below 0.05 was considered statistically significant.

## 3. Results

The population of the Zaanstreek region currently, in 2013, is 143,000; the adherence for endoscopy is even higher, 155,000. This implies that also patients sent for endoscopy are not directly living in the district. Hence, it can be assumed that the number of patients potentially lost to endoscopic follow-up is low.

In the three-year period 2498 colonoscopies were done by two endoscopists. After applying the inclusion (age between 40 and 60 years) and exclusion criteria, 394 (16%) procedures remained for further analysis. The fall of 2013 was used to gather data on the events in these patients in the years following the negative index colonoscopy. Hence, potential follow-up was 19–21 years. In 2013 all included patients (still living) reached an age between 61 and 79 years.

The patients were divided into four groups (Table 1). Group 1 (*n* = 65) included patients who died, group 2 (*n* = 28) patients who were not in the hospital systems anymore nor did they visit the hospital since the index colonoscopy, group 3 (*n* = 136) patients still visiting the outpatient clinics or the hospital but not the department of endoscopy anymore, and finally, group 4 (*n* = 165) patients who underwent one or more new colonoscopies in the years following the index colonoscopy.

Table 2 shows the reason of death in patients from group 1. Two patients died after diagnosis of colorectal cancer: one case of rectal cancer diagnosed 18 years after the index colonoscopy and one case of cecal cancer diagnosed 4 years after the index procedure. Given the time span the cecal cancer could have been based on a missed adenoma, although cecal intubation was successful during the index colonoscopy and an adenoma was not detected. Fourteen patients of group 1 underwent new colonoscopy. The yield of these 14 procedures was a polyp in four patients (two adenomas, one hyperplastic, and one unknown), newly developed diverticuli in two, and colorectal cancer, as already mentioned, in two. Time of death after the index colonoscopy was mean 11.15 years (range 0–20 years).

No data were available from the patients in group 2. This implies either that they moved to another part of the country or another city, or never visited the hospital or the outpatient clinics because of absence of any health issues. Search in the national pathology registry did not reveal any entries on adenomas or colorectal cancer removed in other hospitals after the index colonoscopy.

Patients in group 3 still were consulting specialists in the Zaans Medisch Centrum, internists, cardiologists, surgeons, and so forth but did not undergo new colonoscopy after the index procedure. The fact that these patients still visited their community hospital is strongly suggestive for the assumption that they also would undergo endoscopy, if clinically indicated, in their community hospital.

Patients in group 4 underwent additional colonoscopy. (One additional procedure in 132 patients, 3 procedures in 30 patients, and 4, 5, and 6 colonoscopies in 3 patients resp.) Obviously patients in whom adenomas were detected underwent regular surveillance endoscopy (also included in the above mentioned numbers). In 35 patients polyps were detected (years after the index endoscopy, mean 10.5, range 1–21, and median 10); in the other 130 no polyps were diagnosed (years after the index endoscopy, mean 9.7, range 1–20, and median 10). One polyp appeared to be a leiomyoma; there were 26 adenomas and 3 hyperplastic polyps, and in 5 cases polyps were removed but not sent for histological investigations. In group 4 a total of 3 colorectal cancers were diagnosed, one ascending (20 years after the index procedure) and two cancers in the sigmoid (12 and 13 years after the index procedure).

Five out of 394 patients (1.3%) developed colorectal cancer in the years following the index colonoscopy, while in 39 out of 394 patients (9%) a polyp was detected. Polyps were detected 10.2 years (mean) after the index colonoscopy (standard deviation 5.7, median 10 years).

## 4. Discussion

Colorectal cancer has a high incidence [5, 6]. The disease has a definite precursor lesion, namely, the adenoma. The adenoma-carcinoma sequence is well established and accepted. Removal of adenomas prevents development of cancer [7]. Quality of endoscopy is very important. Small adenomas are easily missed if the colon is cleaned improperly. In addition, the retrieval time during endoscopy is an important factor in detecting adenomas. The adenoma detection rate is higher in endoscopies of high standard [8].

Because the perfect world does not exist, it is stated that many adenomas are missed during colonoscopy in normal daily practice. This especially seems to be the case for small adenomas. The estimates in the literature are around or above 20%. It is stated that this is an enormous problem because these missed adenomas will develop into colorectal cancer and thereby pose an important risk for future health. If indeed so many adenomas are missed, it could be expected that patients with a negative colonoscopy could develop advanced adenomas and ultimately cancer. If this is true, then the cancer incidence in the studied population of 394 patients would be around 80 cases (in 20% of the procedures an adenoma will be missed!). The opposite is true. Only 5 cases of colorectal cancer developed. This is less significant than expected on basis of the literature. It is temptative to assume that many “missed” adenomas never will progress into cancer. Only the case of cecal cancer four years after the index colonoscopy could directly have been attributed to a possibly missed adenoma. Only 39 patients developed a polyp, not all adenomas.

Singh et al. report on 200,857 patients (mean age 74 years, 61% female, 92% white) with a negative colonoscopy. The incidence of colorectal cancer was 1.8 per 1,000 person-years. The incidence was higher in patients >85 years and males [9]. Another study in a cohort of 110,402 individuals with a negative complete colonoscopy, during a 15-year follow-up period, reported on development of 1596 (14.5%) colorectal cancers [10]. In another recent study on the incidence of colorectal cancer in participants of a long-term study, it was shown that the hazard ratios for colorectal cancer were 0.60 (95% CI, 0.53 to 0.68) after negative sigmoidoscopy and 0.44 (95% CI, 0.38 to 0.52) after negative colonoscopy. Negative colonoscopy was associated with a reduced incidence of proximal colon cancer and a reduced incidence of cancer of the distal colon and rectum [11, 12].

Sherer et al. suggested a model framework, in which an individual patient's risk for colonic neoplasia is calculated based on findings from previous colonoscopies. The adjustment of the model predictions operationalizes the clinical knowledge that multiple or advanced neoplasia at baseline colonoscopy is an independent predictor of multiple or advanced neoplasia at follow-up colonoscopy and vice versa for negative colonoscopies and the adjustment of parameter set combination likelihoods accounts for the possibility that patients may have different neoplasia development rates. Such a model could predict the outcome of follow-up colonoscopies [13]. In these population based studies all patients undergoing colonoscopy were included. This implies that also patients with a high risk, like the Lynch syndrome, contributed to the risk.

However, all over the risk of colorectal cancer after a previous negative colonoscopy is reported to be very low. Brenner et al. compared 78 patients with cancers occurring 1–10 years after a negative colonoscopy and 433 colorectal cancers detected at screening. Location in the cecum or ascending colon was independently associated with occurrence of interval cancers. The preceding negative colonoscopy was more often incomplete, indicating that quality of colonoscopy is very important [14].

The present study shows a significant lower number, possibly because the selection bias is less. Patients with a family history of colorectal cancer were excluded from analysis.

This is, to the best of our knowledge, the first study that also looked at adenomas developing after a negative colonoscopy. This number is also rather low. These adenomas could develop into cancer but it seems logical to assume that many patients will not have the life span anymore to get colorectal cancer.

If the perfect world would exist then no adenomas were missed and many patients would have been included in surveillance programs and would undergo many follow-up colonoscopies with only little clinical advantages and many possible complications of the procedures.

Of course the present study has several flaws. But on the other hand this is the best there is. It is not a prospective follow-up with colonoscopies at predefined intervals. Many people and patients could have developed adenomas, in the meanwhile, that go undetected because no colonoscopy was done. But on the other hand, these newly developed or missed adenomas obviously did not pose a clinical problem. Occult development of adenomas did not lead to rectal bleeding or other symptoms. Thus, the question can be posed: how important is it to detect polyps that probably never will lead to a clinical problem? It can be expected that if a patient develops colorectal cancer due to a missed adenoma in the past, this will not go clinically unnoticed.

Given the results of the present study, in the light of all its shortcomings, it probably is clear that it is not the effort to detect and remove every small adenoma. Possibly these efforts will lead to overtreatment and potential complications. Of course, there is no discussion in detecting even small adenomas in patients with a family history or the Lynch syndrome. Every adenoma should be removed in these patients.

It can be concluded from the present study that the potential miss rate of adenomas does not have major implications for future health and that the potential implications of missed adenomas are highly exaggerated. The only exception is patients with the Lynch syndrome who have a high risk of developing interval cancers.

## Figures and Tables

**Table 1:** The demographics, findings, and cecal intubation rate in the four groups of patients.

	Group 1	Group 2	Group 3	Group 4	
Men	31	15	56	60	*P* = ns
Women	34	13	80	105	
Diverticuli	19	3	20	30	*P* = ns
Haemorrhoids	10	5	22	23	
Long colon	10	1	10	21	
Cecal intubation (%)	70	86	79	82	

**Table 2:** Reason for death in patients of group 1.

Cardiovascular	11 (17%)
Pulmonary	3 (4.6%)
Cancer (no colorectal cancer)	19 (29%)
Miscellaneous	9 (24.4%)
Unknown	21 (32%)
